# Cadmium nanocluster as a safe nanocarrier: biodistribution in BALB/c mice and application to carry crocin to breast cancer cell lines

**DOI:** 10.37349/etat.2024.00233

**Published:** 2024-05-28

**Authors:** Moslem Jafarisani, S. Ali Hashemi, Nassim Faridi, Mir F. Mousavi, S. Zahra Bathaie

**Affiliations:** Changchun Institute of Applied Chemistry, Chinese Academy of Sciences, China; ^1^Department of Clinical Biochemistry, Faculty of Medical Sciences, Tarbiat Modares University (TMU), Tehran 14155-331, Iran; ^2^Institute for Natural Products and Medicinal Plants (INPMP), Tarbiat Modares University (TMU), Tehran 14155-331, Iran; ^3^Department of Chemistry, Faculty of basic Sciences, Tarbiat Modares University (TMU), Tehran 14115-175, Iran

**Keywords:** Cadmium nanocluster, breast cancer cell line, hyaluronic acid, CD44, oxidative stress, antioxidant enzymes

## Abstract

**Aim:**

Metal nanoclusters are emerging nanomaterials applicable for drug delivery. Here, the toxicity and oxidative stress induction of divalent cationic cadmium (Cd^2+^) was compared with a Cd in the form of nanocluster. Then, it was used for targeted drug delivery into breast cancer cell lines.

**Methods:**

Using a green chemistry route, a Cd nanocluster (Cd-NC) was synthesized based on bovine serum albumin. After characterization, its genotoxicity and oxidative stress induction were studied in both *in vitro* and *in vivo*. After that, it was conjugated with hyaluronic acid (HA). The efficiency of hyaloronized-Cd-CN (HA-Cd-NC) for loading and releasing crocin (Cro), an anticancer phytochemical, was studied. Finally, it was applied for cell death induction in a panel of breast cancer cell lines.

**Results:**

The comet assay results indicated that, unlike Cd^2+^ and potassium permanganate (KMnO_4_), no genotoxicity and oxidative stress was induced by Cd-NC *in vitro*. Then, the pharmacokinetics of this Cd-NC was studied *in vivo*. The data showed that Cd-NC has accumulated in the liver and excreted from the feces of mice. Unlike Cd^2+^, no toxicity and oxidative stress were induced by this Cd-NC in animal tissues. Then, the Cd-NC was targeted toward breast cancer cells by adding HA, a ligand for the CD44 cell surface receptor. After that, Cro was loaded on HA-Cd-NC and it was used for the treatment of a panel of human breast cancer cell lines with varying degrees of CD44. The half-maximal drug inhibitory concentration (IC_50_) of Cro was significantly decreased when it was loaded on HA-Cd-NC, especially in MDA-MB-468 with a higher degree of CD44 at the surface. These results indicate the higher toxicity of Cro toward breast cancers when carried out by HA-Cd-NC.

**Conclusions:**

The Cd-NC was completely safe and is a promising candidate for delivering anticancer drugs/phytochemicals into the targeted breast tumors.

## Introduction

Targeted drug delivery is an emerging area of research aimed at increasing the efficacy of medications. This system carries and delivers a drug (an anticancer drug) to the targeted tissue (tumor). As a result, the drug accumulates in the diseased area. This strategy reduces the toxicity and side effects of the medications for other tissues. It directly releases the drug into the target tissue improving efficiency and reducing the required concentration [1]. By employing multidisciplinary field benefits of research, such as nano-structured materials [2] including nanoparticles (NP) [3], nanoclusters [4], and nanofibers [5], outstanding achievements have been made in the field of targeted drug delivery. Functionalization is another appealing strategy for improving the capability of nanomaterials [6]. Many benefits have been suggested, such as increasing the rate of their circulation in the body, targeting specific cell surface receptors, and increasing the permeability of the target cell membrane [7].

Among the nanomaterials, metal nanoclusters are a relatively new nanocarrier class that has attracted much attention for their unique properties [4, 8]. They were used as nanocarriers of some anticancer drugs, fluorescent probes in cellular imaging [9–11], and nano-biosensor [10]. Combining the specific ligands against cancer cell surface receptors in these nanoclusters can also be applied in targeted drug delivery systems [12]. Following the previous works, a cadmium nanocluster (Cd-NC) was synthesized based on bovine serum albumin (BSA, Cd-NC@BSA). As a capping agent, BSA converts Cd^2+^ to the metallic Cd atom and then to the nanocluster (Cd-NC) [9]. Therefore, its biocompatibility and safety was studied both *in vitro* and *in vivo* before it was applied for delivering the anticancer compound to a panel of breast cancer cell lines.

The anticancer effect of phytochemicals has been reviewed extensively [13–15]. Crocin (Cro), the main carotenoid of saffron (*Crocus sativus* L.), has been introduced as an anticancer compound [15–19]. The half-maximal drug inhibitory concentration (IC_50_) of Cro has been reported between 2 μmol/mL to 5.5 mmol/mL in different cancers [15, 20, 21]. A recent mechanistic study confirmed its role in inducing oxidative stress in breast cancer cells, followed by apoptotic cell death induction [21]. Herein, in addition to investigating the safety/toxicity of the Cd-NC as a nanocarrier in both *in vitro* and *in vivo* conditions, it was targeted by adding hyaluronic acid (HA). Then Cro loading and releasing on it was investigated. Finally, the anticancer efficacy of free Cro and Cro-loaded in this Cd-NC was studied and compared in a panel of breast cancer cell lines with different degrees of CD44 cell surface receptor.

## Materials and methods

### Instrumentation

The following instruments were used in this study. Water bath-type Ultrasonicator (EngoTech, Zurich, Switzerland). Ultraviolet (UV) visible (UV-vis) absorption measurements were performed with a double-beam Schimadzu-3100 spectrophotometer (Kyoto, Japan). Spectrofluorimetric measurements were performed using a Shimadzu Model RF-5000 spectrofluorometer (Kyoto, Japan). Far-UV circular dichroism (CD) spectra were obtained using a J-810 spectropolarimeter (JASCO, Tokyo, Japan). The ζ-potential and hydrodynamic diameter of the Cd-NCs were measured by dynamic light scattering (DLS) using a zeta sizer Nano ZS instrument (Malvern, Worcestershire, UK). For Fourier transform infrared (FT-IR) spectroscopy, samples were lyophilized, mixed with KBr (Sigma Chem. Co. St Louis, USA) to make pellets, and analyzed using a Perkin-Elmer spectrophotometer (Perkin-Elmer Inc., USA). The scanning electron microscopy (SEM) was done using a TESCAN VEGA//XMU and TESCAN’s Essence™ software (Kohoutovice, Czech Republic). The flame atomic absorption spectroscopy (FAAS, AA-7000, Shimadzu, Kyoto, Japan) was used to determine the Cd in samples. Sonicator-3000 (Misonix, Farmingdale, NY, USA) was used to homogenize tissue samples.

### Synthesis of the nanocluster

The Cd-NC was synthesized using a modified method described previously [9]. Briefly, the BSA solution (Sigma Chem. Co. St Louis, USA), 20 mg/mL in distilled water (DW), was gradually mixed with the same volume of cadmium chloride (CdCl_2_, 5 mmol/L, Merck Co., Germany), on a stirrer. After 1 min, the pH was gradually increased to 13 by adding the NaOH (1 mol/L, Sigma Chem. Co. St Louis, USA). Then the temperature was increased to 45℃. After 7 h, the solution was sonicated for 1 h, with 5–10 min intervals, using a Bath Sonicator. After that, it was dialyzed using a dialysis bag [molecular weight cut off (MWCO): 12 kDa] against phosphate buffer saline (PBS). After 72 h, it was dried using a freeze dryer (Eyela, FDU-1200) and retained at –20℃ until use.

In the next step, HA [5 mg/mL in DW, sodium hyaluronate, molecular weight (MW) = 100–120 kDa was obtained from Lifecore Biomedical] was functionalized using 1-ethyl-3-(3-dimethylaminopropyl)-carbodiimide (EDC, Sigma Chem. Co. St Louis, USA) and *N*-hydroxysuccinimide (NHS, Sigma Chem. Co. St Louis, USA), and were added to the Cd-NC solution, resulting in hyaloronized-Cd-NC (HA-Cd-NC) formation. Then, different methods, including UV-vis spectroscopy, fluorescence, DLS, FT-IR, CD, and SEM were used to characterize the formation of both Cd-NC and HA-Cd-NC.

### Loading and releasing of Cro

Cro was extracted and purified from saffron (*Crocus Sativus* L.) according to the previously approved method [22]. Briefly, the experimental procedure for Cro loading on HA-Cd-NC was as follows. HA-Cd-NC powder (1 mg) was mixed with different amounts of Cro (0.1 mg, 0.2 mg, 0.3 mg, 0.4 mg, and 0.5 mg) and dissolved in PBS. The final volume was adjusted to 1 mL and then gently stirred in the dark for 24 h in a shaker incubator. After that, it was centrifuged at 15,000 *g* for 10 min. The supernatant was separated, and the pellet was mixed with 1 mL PBS and centrifuged. The procedure was repeated two times until a colorless supernatant was obtained. The supernatants of three times repeats were mixed (3 mL). The pellet containing Cro-HA-Cd-NC@BSA (Cro-NC) was freeze-dried and stored at –20℃ for further analysis and application. The percentages of the Cro encapsulation efficiency (EE) were estimated using Equation 1 [23].

EE (%) = W_Cro-Cd-NC_/W_Cro_ × 100

Where W_Cro-Cd-NC_ is the amount of Cro loaded on this nanocluster and W_Cro_ is the total (initial) amount of Cro was added to HA-Cd-NC.

To evaluate the percentage of the EE of Cro, at each W:W ratio of Cro:NC, the absorbance of the supernatants obtained from the previous steps containing free Cro was read at 440 nm, and using a standard curve of Cro, the concentration of free Cro (unbound) was calculated and subtracted from the initial amount (W_Cro-cd-NC_ = W_Cro_ – W_free Cro_). The remaining was the amount of bound Cro (loaded in the HA-Cd-NC), and using Equation 1, the EE percentage (EE%) of Cro was calculated.

The Cro releasing test was conducted under both physiological and acidic pH (7.4 and 5.3, respectively). For this purpose, the dried HA-Cd-NC containing Cro (0.5 mg) was weighed and dissolved in 2 mL PBS, pH = 7.4 or pH = 5.3, and mixed for 10 min, using a Vortex. The resulting clear solution was transferred into a 12 kDa dialysis tube, immersed in 10 mL PBS, pH = 7.4 or pH = 5.3, and placed in a shaker incubator at 37℃, in the dark. Then, at different time intervals, the absorbance of 2 mL buffer was read at 440 nm. This buffer was returned to the Becker to avoid the volume change. This process was completed in up to 24 h. Free Cro released from a similar dialysis tube was applied as a control.

### *In vitro* toxicity and oxidative stress assay

Since Cd^2+^ is highly toxic and has been known as a potent oxidative stress inducer, its toxicity after incorporation into the NC should be investigated. For this purpose, the cytotoxicity studies were performed in dose- and time-dependent manners. At first, the genomic toxicity of Cd-NC was investigated by the comet assay on HeLa cells as a human cancer cell line other than breast cancer. The results were compared with the toxicity of free Cd ions in the form of CdCl_2_ and KMnO_4_. The cells with no treatment were also used as controls.

Comet assay or single-cell gel electrophoresis (SCGE) was performed according to the method described previously [24, 25]. Briefly, HeLa cells were cultured in appropriate numbers in 6-well flasks and Dulbecco’s Modified Eagle Medium (DMEM-F12), high glucose medium containing 2 mmol/L *L*-glutamine (Sigma Chem. Co. St Louis, USA), 10% FBS and 1% pen/strep (Life Technology, Paisley, UK). Then, they were treated with different concentrations of CdCl_2_ (150 μmol/L, 200 μmol/L, and 300 μmol/L), Cd-NC (0.1 mg/mL, 0.5 mg/mL, and 1 mg/mL), and KMnO_4_ (250 μmol/L). The control group received no more treatment. After 24 h and providing the appropriate number, the cells were trypsinized. Live cells were counted using trypan blue staining, and the images were analyzed using Image J software (version 1.49t, NIH approved) and a Comet analyzer (CAA-500).

In the second step, oxidative stress parameters such as malondialdehyde (MDA) and glutathione (GSH) concentrations and the specific activity of some antioxidant enzymes [GSH peroxidase (GPX), catalase (CAT), and superoxide dismutase (SOD)] were determined in the HeLa cells. MDA (lipid peroxidation colorimetric/fluorometric assay kit, K739, Biovision, USA) and GSH were measured by commercial kits (GSH colorimetric assay kit, K261, Biovision, USA) on cellular extracts of the cells in different groups. The GPX and CAT activities were measured by the GPX assay kit (K762-100 Biovision, USA) and CAT activity colorimetric/fluorometric assay kit (K773-100, Biovision, USA). The SOD activity was measured by the SOD assay kit (7500-100-K, Funakoshi, Japan). The protocols were performed according to the manufacturer’s kit. The protein concentration of all samples was determined using the Bradford method [26], and the specific activity of the enzymes was determined by dividing the enzyme unit per mg of protein.

### *In vivo* toxicity and oxidative stress assay

The *in vivo* biodistribution of Cd-NC and the possibility of its toxicity were investigated in a murine model. For this purpose, twenty female BALB/c mice, eight weeks old were purchased from Pasture Institute, Karaj, Iran. Animals were kept under ad libitum access to water, an ordinary diet, and a 12 h/12 h light/dark cycle in the Tarbiat Modares University (TMU) Animal Laboratory. The animal care protocol and use were based on the guidelines of laboratory animals prepared by the TMU. After two weeks of acclimation, mice were entered into a 30-day study. Thus, they were randomly divided into four groups (5 in each) and named as follows:

(1) D0: control group with no treatment. They received 100 μL of PBS through intra-peritoneal (i.p.) injection on 1st day of the study and then sacrificed on the 30th day.
(2) D1: they were treated with 100 μL of Cd-NC (1 mg/mL) by i.p. injection on the 29th day of study and then sacrificed on the 30th day. Thus, they were only exposed to the NC for one day.
(3) D7: this group was treated with 100 μL of Cd-NC (1 mg/mL), by i.p. injection on the 23rd day of the study, then sacrificed on the 30th day. This group was exposed to this NC for seven days.
(4) D30: they were treated with 100 μL of Cd-NC (1 mg/mL) by i.p. injection on 1st day of study and then sacrificed on the 30th day. Thus, they were exposed to the Cd-NC for thirty days.

The body weight of all groups was determined weekly. Before sacrificing, the samples of urine and feces were collected by the previously described method [27]. Then, the mice were weighed, and after anesthesia with ketamine/xylazine, their blood was collected via cardiac puncture. Major organs, including the liver, kidney, spleen, lung, and heart were removed, washed with PBS, and weighed. A section of each tissue was prepared and kept in neutral buffered formalin (4%) for hematoxylin and eosin (H & E) staining. The remaining was kept at –80℃ for further experiments. The body indices were calculated as the organ weight/total body weight ratio. Pathological examination with a digital microscope was done by a specialist in the field.

The blood and urine samples were prepared for Cd determination, with some modifications of the previously described method [28]. Briefly, blood or urine samples (50 μL) were mixed with 200 μL DW. Then, hydrochloric acid (10%, 500 μL, Merck Co., Germany) was added and the samples were analyzed using a FAAS.

The feces samples were defrosted, weighted, and digested in 70% nitric acid (Sigma Chem. Co. St Louis, USA). The solution was heated to evaporate the water, and then the remnant was homogenized in 4.5 mL DW. Then, 500 μL hydrochloric acid 10% was added. All samples were analyzed in duplicate, using a FAAS [29].

The tissue samples were weighted and homogenized in PBS using a Sonicator-3000. The resulting mixture was centrifuged at 3000 *g* for 10 min. The supernatant was used for determination of protein content using the Bradford method [26], as well as the CAT [30], SOD [31], and GSH *s*-transferase (GST) activities [32]. In addition, MDA and GSH content were measured using previously explained methods [33, 34]. All parameters were presented as values per mg protein of the tissue sample.

### Viability assay in a panel of breast cancer cell lines

At this stage, the viability of four breast cancer cell lines (MDA-MB-468, MDA-MB-231, BT-474, and MCF-7) was investigated and compared in the presence of free Cro, Cd-NC, HA-Cd-NC, and Cro-NC using the 3-(4,5-dimethylthiazol-2-yl)-2,5-diphenylte-tetrazolium bromide (MTT) assay, as we described recently [21].

### Statistical Analysis

Data are expressed as the mean ± standard error of mean of at least three independent repeats. Statistical analysis was performed using SPSS 16.0 (SPSS Inc., Chicago, IL, USA). The data were analyzed using an unpaired *t*-test or one-way analysis of variance (ANOVA). Statistical significance for all tests was set at 95% confidential limits, *P* < 0.05.

## Results

### Synthesis of Cd-NC

**Figure 1:**
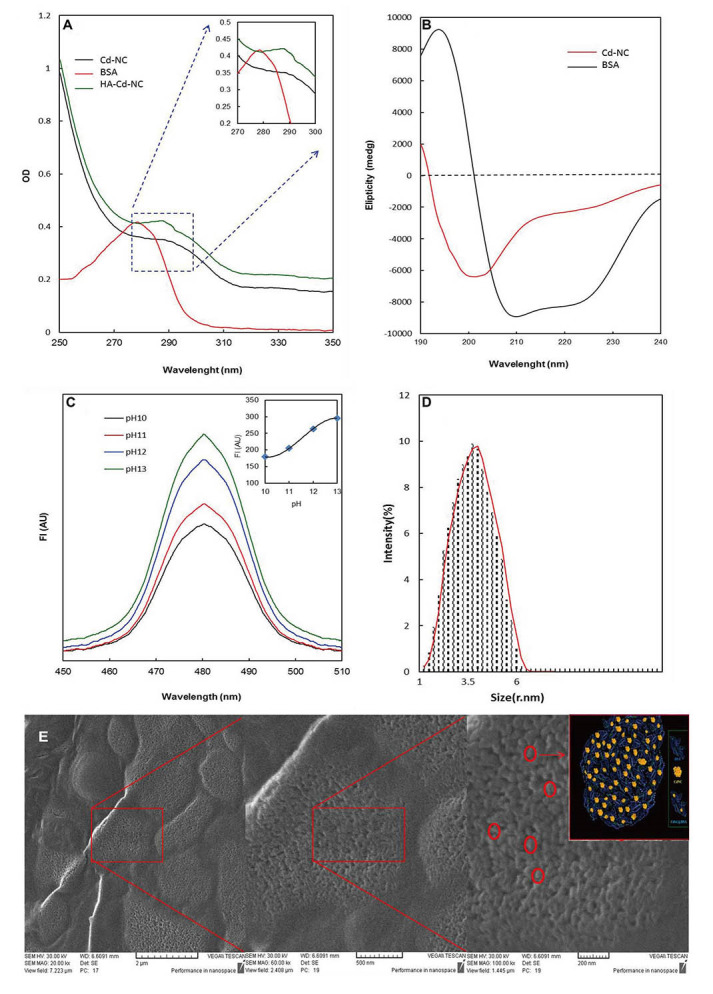
Spectroscopic and microscopic characterization of the nanocluster. (A) Changes in the BSA absorption at different stages of nanocluster synthesis; (B) the CD spectrum of free BSA and BSA templated Cd-NC; (C) quantitative evaluation of the FI of nanocluster formation at different pH; (D) nanocluster size was determined by zeta-sizer indicating that the particle size was in the range of 1 nm to 6 nm, with the highest density in the range of 3.5 nm; (E) the SEM image of the Cd-NC. In this figure, the decrease at the 280 nm peak and the addition of the peak at 290 nm are characteristic of the cadmium nanocluster structure (Figure 1A). The inset shows the changes in the specific area on a larger scale (Figure 1A). The decrease in the peak intensity at 208 nm and 220 nm indicates that the helix content of BSA has decreased due to the Cd-NC formation (Figure 1B). In Figure 1C, with increasing pH, the number of atoms involved in the formation of cluster state increases, and therefore, the fluorescence emission increases. The excitation wavelength in this study was 360 nm. The inset indicates that the FI reaches a plateau at pH = 13, which used in this study. In Figure 1E, the figures from left to right show different magnifications of 2 μm, 500 nm, and 200 nm, respectively. The contents of the red circles are shown schematically in the inset of the right panel. A.U.: arbitrary unit

The Cd-NC was synthesized and targeted toward the breast cancer cells by adding HA. The characteristic peaks of Cd-NC at pH = 13 were plotted using spectrophotometry (Figure 1A), CD (Figure 1B), and fluorescence intensity [FI, (Figure 1C)]. The FI of the complex was increased by increasing pH up to 13 (Figure 1C), which is the best situation for forming the Cd-NC. The plot obtained by Zeta Sizer is shown in Figure 1D, indicating the nano size of the Cd-NC. The SEM images (Figure 1E) indicates that the Cd-NC coated with a layer of gold. The images are shown at different magnifications, indicating the Cd-NC’s nano size.

### *In vitro* safety and toxicity assay

**Figure 2:**
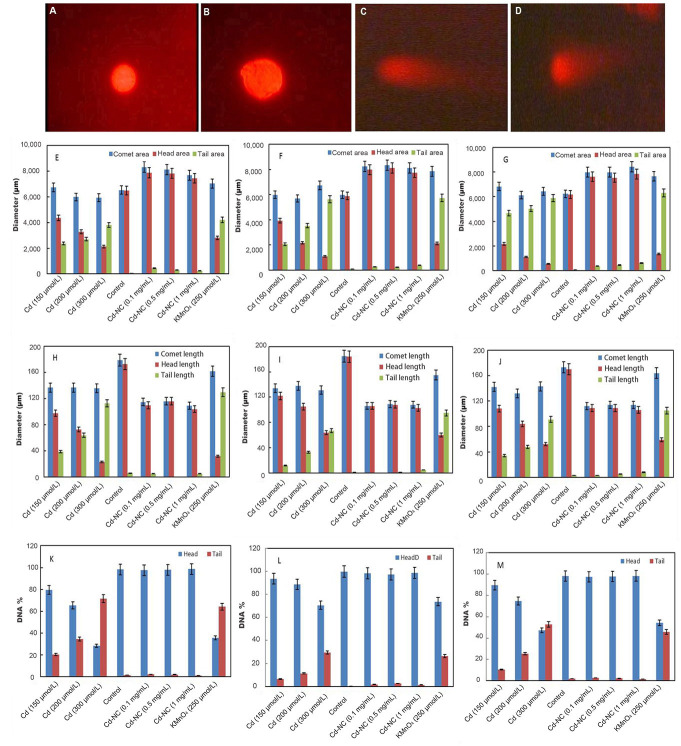
The fluorescence images of HeLa cells in a comet assay and analysis of the results after incubation at different intervals. (A) HeLa cells with no treatment; (B) HeLa cells treated with Cd-NC; (C) HeLa cells treated with KMnO_4_; (D) HeLa cells treated with CdCl_2_; (E) the comet, head, and tail areas at 12 h after treatment; (F) the comet, head, and tail areas at 24 h after treatment; (G) the comet, head, and tail areas at 48 h after treatment; (H) the changes in the comet, head, and tail lengths at 12 h after treatment; (I) the changes in the comet, head, and tail lengths at 24 h after treatment; (J) the changes in the comet, head, and tail lengths at 48 h after treatment; (K) the DNA% in the head and tail areas of the comet in 12 h after treatment of different groups; (L) the DNA% in the head and tail areas of the comet in 24 h after treatment of different groups; (M) the DNA% in the head and tail areas of the comet in 48 h after treatment of different groups. The magnification of Figure 2A–D was 400. A dose-dependent increase is observed in the tail area of the Cd^2+^ treated group (Figure 2E–G). The statistical analysis indicates significant changes in the highest doses of CdCl_2_ at *P* < 0.003 after 12 h and *P* < 0.001 after 24 h and 48 h compared to the control group (Figure 2E–G). A dose-dependent increase in the tail length of the Cd^2+^ treated group is observed (Figure 2H–J). The statistical analysis indicates significant changes in the highest dose of CdCl_2_ compared to the control at *P* < 0.001 at 12 h, 24 h, and 48 h (Figure 2H–J). A time- and concentration-dependent increase in the DNA% in the tail of the Cd^2+^ treated cells is observed (Figure 2K–M). The statistical analysis indicates significant changes in the highest dose of CdCl_2_ compared to the control at *P* < 0.003, *P* < 0.001, and *P* < 0.001 at 12 h, 24 h, and 48 h, respectively (Figure 2K–M)

The fluorescence microscopy images of the HeLa cells in the absence of any treatment (Figure 2A) and after 12 h incubation with Cd-NC (Figure 2B), KMnO_4_ (Figure 2C), or CdCl_2_ (Figure 2D) are shown in Figure 2. In this experiment, KMnO_4_ was used as a positive control, and CdCl_2_ was used to compare the effect of Cd in the atomic and ionic forms. In contrast to Figure 2B, Figure 2C and 2D show significant changes and tail formation, which are the characteristics of DNA damage in eukaryotic cells.

The comet, head, and tail areas (at 12 h, 24 h, and 48 h) are shown in Figure 2E–G, respectively, in different concentrations of Cd^2+^ or equivalent amounts of Cd-NC, and KMnO_4_. The results indicate significant differences in the groups treated with Cd^2+^ or KMnO_4_ compared to the control. For more clarity, the ratio of the head area/tail area in each condition was calculated and represented in Table 1. This table also clearly indicates differences between these ratios in different groups. There were no significant changes in these ratios between groups treated with the equivalent amounts of Cd-NC compared to the control group.

**Table 1:** The ratio of tail/head of comet parameters obtained from the data of Figure 2E to 2M.

**Time**	**Group name**	**Tail area/Head area**	**Tail length/Head length**	**Tail DNA%/Head DNA%**
12 h	Control	0.01	0.01	0.00
	KMnO_4_ (250 µmol/L)	1.5	1.58	0.36
	Cd (150 µmol/L)	0.54	0.1	0.07
	Cd (200 µmol/L)	0.82	0.31	0.13
	Cd (300 µmol/L)	1.79	1.05	0.42
	Cd-NC (0.1 mg/mL)	0.06	0.01	0.02
	Cd-NC (0.5 mg/mL)	0.04	0.01	0.03
	Cd-NC (1 mg/mL)	0.03	0.05	0.01
24 h	Control	0.01	0.02	0.02
	KMnO_4_ (250 µmol/L)	2.7	1.78	0.84
	Cd (150 µmol/L)	0.52	0.31	0.12
	Cd (200 µmol/L)	1.63	0.57	0.34
	Cd (300 µmol/L)	5.13	1.75	1.11
	Cd-NC (0.1 mg/mL)	0.03	0.03	0.03
	Cd-NC (0.5 mg/mL)	0.03	0.05	0.02
	Cd-NC (1 mg/mL)	0.05	0.07	0.02
48 h	Control	0.01	0.04	0.02
	KMnO_4_ (250 µmol/L)	4.69	4.06	1.79
	Cd (150 µmol/L)	2.15	0.4	0.26
	Cd (200 µmol/L)	4.6	0.88	0.53
	Cd (300 µmol/L)	10.87	4.91	2.53
	Cd-NC (0.1 mg/mL)	0.05	0.04	0.02
	Cd-NC (0.5 mg/mL)	0.06	0.01	0.02
	Cd-NC (1 mg/mL)	0.08	0.05	0.01

Changes in the comet, head, and tail lengths at 12 h, 24 h, and 48 h, respectively, under different conditions (Figure 2H–J). These figures and analysis of the data in Table 1 also indicate significant changes in the head-to-tail ratios in the presence of either Cd^2+^ or KMnO_4_ compared with the control and Cd-NC treated cells. There were also no significant changes in these parameters due to Cd-NC treatment. Although there were no significant changes in the tail length after Cd-NC treatment of the cells, it was increased up to 5,890 µmol/L and 6,308 µmol/L (more than 10 times) after 48 h of 300 µmol/L of Cd and KMNO_4_ treatment, respectively.

After separating the head and tail sections, the genomic DNA content was determined and analyzed in each part to estimate the degree of DNA damage in terms of DNA strand break. These parameters are represented by the DNA% at each segment. The DNA% at 12 h, 24 h, and 48 h and at different conditions are shown in Figure 2K–M. The results indicate a significant and time-dependent increase in the DNA% in the tail segments of the cells treated with Cd^2+^ compared to the control cells at 48 h. The DNA% in the tail was increased more than 50 times after 48 h treatment of the cells with higher Cd concentration or KMNO_4_ compared with the Cd-NC.

**Figure 3:**
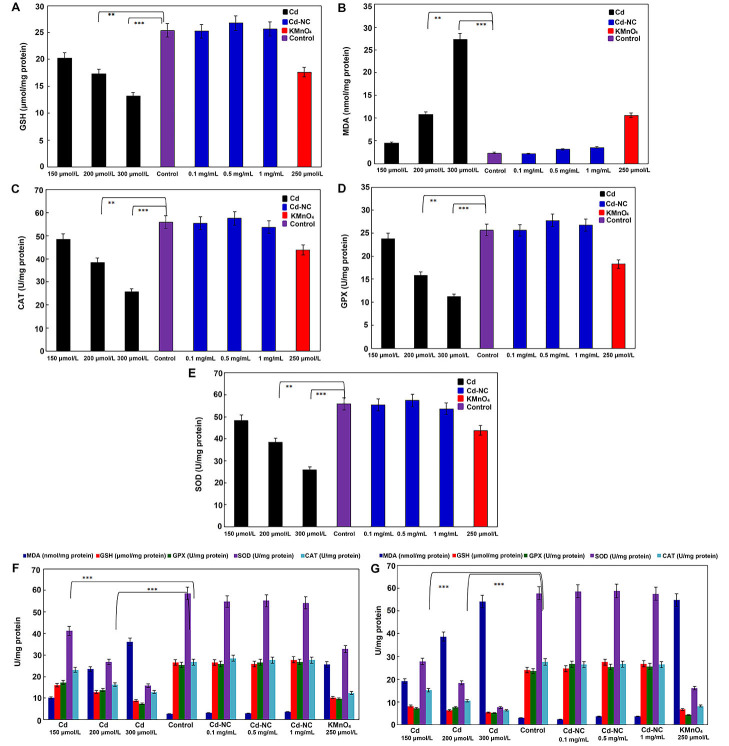
The effect of different concentrations of KMnO_4_, free Cd, and Cd-NC on the oxidative stress parameters in HeLa cells. (A) GSH in HeLa cell line, after 12 h incubation; (B) MDA in HeLa cell line, after 12 h incubation; (C) CAT in HeLa cell line, after 12 h incubation; (D) GPX in HeLa cell line, after 12 h incubation; (E) SOD in HeLa cell line, after 12 h incubation; (F) the changes in all the parameters (GSH, MDA, CAT, GPX, and SOD) after 24 h; (G) the changes in all the parameters (GSH, MDA, CAT, GPX, and SOD) after 48 h. KMnO_4_ was used as a standard oxidant. ^**^ and ^***^ indicate statistically significant differences between the control group and the named CdCl_2_ concentration at *P* < 0.01 and *P* < 0.001, respectively

The data presented in Figure 3A and 3B indicate that after 12 h of incubation, higher concentrations of Cd^2+^ caused a more significant decrease in the GSH and a more significant increase in the MDA concentrations, respectively. Significant decreases in the specific activities of the antioxidant enzymes (CAT, GPX, and SOD, respectively) were observed in the dose- and time-dependent manners due to the treatment of cells with either Cd^2+^ or KMnO_4_ (Figure 3C–E). At the same time, the Cd-NC with similar loading concentrations of Cd^2+^ did not affect the specific activity of these enzymes.

As observed in Figure 3F and 3G, the observed changes in all parameters were continued up to 24 h and 48 h of treatments, indicating the time-dependent alterations. However, the Cd-NC had no significant effect on these parameters at any time or at equivalent concentrations.

### Biodistribution, *in vivo* safety, and toxicity assay

**Figure 4:**
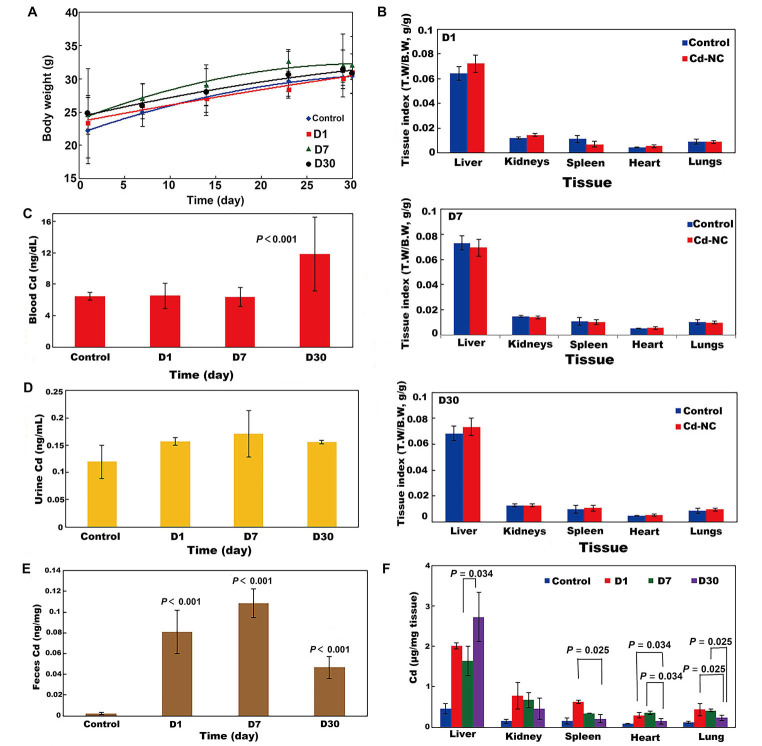
The effect of Cd-NC on body parameters in mice. (A) Body weight; (B) body indexes in different groups of D1, D7, and D30 in this study; (C) biodistribution of Cd-NC at the end of the experiment in different mice groups (D1, D7, and D30) in the blood; (D) biodistribution of Cd-NC at the end of the experiment in different mice groups (D1, D7, and D30) in the urine; (E) biodistribution of Cd-NC at the end of the experiment in different mice groups (D1, D7, and D30) in the feces; (F) Cd µg/mg tissue in different groups, at different time intervals. Statistically significant changes between parameters, if exist, are shown in the Figure 4C–E. The vertical axis in Figure 4B show the tissue weight to body weight ratios (T.W/B.W, both in grams) in different groups at different times. There were no significant differences between these parameters in the control and other groups (Figure 4B)

The weekly changes in the body weight of all mouse groups during the experimental period are shown in Figure 4A. It indicates no significant changes in the body weight of animals in different groups. The tissue indexes (the ratio of tissue weight/ body weight) of the animal groups at the end of the experimental period are seen in Figures 4B (D1, D7, D30). These indicate no significant changes in the animal tissues after treatment with Cd-NC compared to the control group. It means that treatment of the mice with this Cd-NC did not cause any adverse effect on the growth and body weight of animals in the experiment period. In addition, no mortality, abnormal clinical signs, and behaviors were observed in the animals.

The biodistribution of Cd-NC into mice’s blood, urine, and feces, after 1 day, 7 days, and 30 days of administration are shown in Figure 4C–E. Considering the basal level of Cd in the blood of the control animals (Figure 4C), there were no significant changes after 1 day and 7 days of Cd-NC treatment, but the blood concentration of Cd significantly increased after 30 days. In animals’ urine and feces (Figure 4D and 4E), Cd increased significantly until day seven and then decreased. However, changes in the feces Cd concentration were more than in the urine.

**Figure 5:**
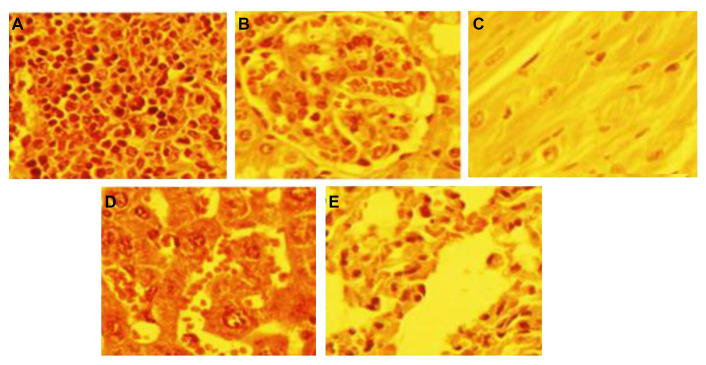
The pathology of different organs of mice treated with Cd-NC. (A) Liver; (B) kidney; (C) heart; (D) spleen; (E) lung. There are no significant changes in the tissues compared with the normal mouse tissues [58]. Staining with (H & E), magnification × 400

The biodistribution of Cd in various tissues of different groups after sacrificing the animals at the end of the experiment are shown in Figure 4F. It indicates that the accumulation of Cd was more in the liver. The microscopic investigation of these tissues is shown in Figure 5, it indicates no significant pathologic changes in the Cd-NC-treated mice’s liver, kidney, spleen, lung, and heart tissues after 30 days (Figure 5A–E), unless there are some little changes in the liver tissue (Figure 5A).

**Figure 6:**
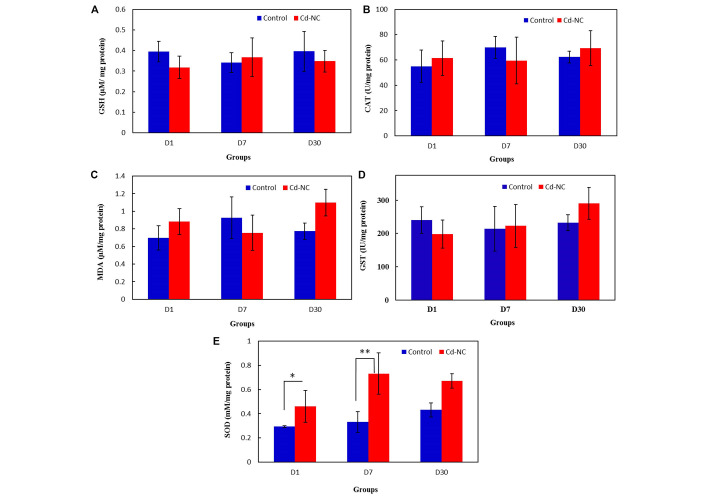
The effect of Cd-NC on the oxidative stress parameters. (A) GSH; (B) MDA; (C) antioxidative enzymes CAT in the Cd-NC treated mice compared with a control group with no treatment; (D) antioxidative enzymes GST in the Cd-NC treated mice compared with a control group with no treatment; (E) antioxidative enzymes SOD in the Cd-NC treated mice compared with a control group with no treatment. There were only statistically significant differences observed between the specific activity of SOD in the liver of the control group with the group treated with Cd-NC at 1 day and 7 days, ^*^ *P* < 0.006 and ^**^ *P* < 0.0001. The increasing trend of this parameter after 30 days, was not statistically significant

The data in Figure 6A and 6B show the amount of GSH and MDA in the livers of the control and Cd-NC-treated mice in all groups. The specific activities of CAT and GST in the livers of the control and Cd-NC-treated mice are shown in Figures 6C and 6D. These data indicate no significant changes in these values between the control and Cd-NC-treated mice. However, a significant increase in SOD-specific activity is observed in the liver after 1 day and 7 days of Cd-NC administration (Figure 6E). Although an increasing trend was also seen after 30 days, it was not statistically significant.

#### Application in targeted-drug delivery

**Figure 7:**
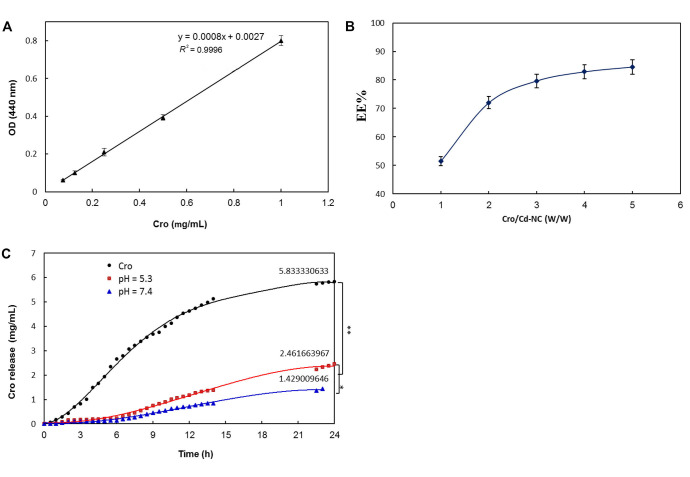
The efficiency of Cro loading and releasing of Cd-NC. (A) Cro standard curve; (B) the EE percentage of Cro in the Cd-NC, which was calculated using Equation 1; (C) the percentage of Cro releasing from Cd-NC at different pH. The black plot is the free Cro as a control. The red and green plots show Cro released from Cd-NC at acidic and neutral pH. The results show a significant difference (^**^ *P* < 0.003 at 24 h) between releasing free and encapsulated Cro from dialysis tubes. Also, the pH changes caused a significant difference (^*^ *P* < 0.01 at 24 h) in Cro-releasing potency

After ensuring the safety of the Cd-NC for human cell line and mouse, it was targeted with HA and then loaded with Cro as an anticancer compound. The calibration curve of Cro concentrations is shown in Figure 7A, and the results of Cro loading on HA-Cd-NC are shown in Figure 7B. The Cro releasing from the dialysis tube (black) and Cd-NC at acidic pH (red) and neutral pH (green) is shown in Figure 7C. This figure indicates more Cro releasing at acidic pH (cancer cell environment) than at neutral pH.

**Figure 8:**
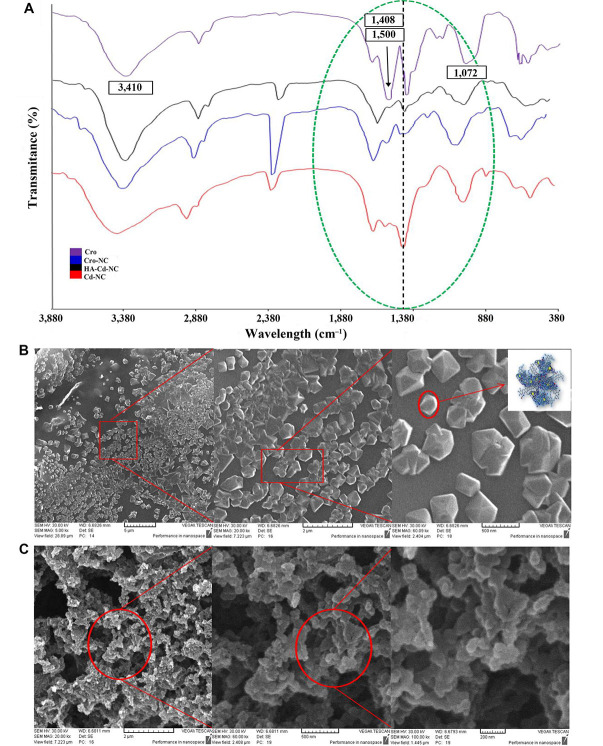
The characteristics of Cd-NC after Cro loading. (A) The FT-IR results of the Cd-NC after HA and Cro addition compared with the spectrum of Cro alone; (B) the SEM image of the Cd-NC after adding hyaluronic acid in the 5 µm, 2 µm, and 500 nm scale and a schematic model for HA-Cd-NC in the right panel; (C) the SEM image of Cd-NC after Cro loading and the scales are 2 µm, 500 nm, and 200 nm from left to right, respectively. In Figure 8A, the characteristic peaks of Cro are shown in the figure. Peaks around 3,410 cm^–1^, characteristics of OH groups, with some shifts are observed in all compounds. The dotted black line shows the characteristic peak of Cd-NC, which was shifted after the addition of some other components. All critical peaks in this region are seen in the region surrounded by a green dotted line

The characteristics FT-IR peaks of Cro before and after entrapment in HA-Cd-NC and Cd-NC are shown in Figure 8A. A bisignate signal around 1,408 cm^–1^ and 1,500 cm^–1^ of Cro was changed in the Cro-NC, indicating its incorporation in this nanocarrier. There are some peaks of both Cro and HA-Cd-NC, overlapped after conjugation (e.g., the peak at 1,702 cm^–1^), suggesting the presence of Cro with its functional groups, with no significant changes within the nanocluster in the conjugate. The SEM images of HA-Cd-NC before and after Cro loading are shown in Figure 8B and 8C. It indicates that the HA provides a cover for Cd-NC, which increases the nanocarrier biocompatibility. The observed change in the morphology of the Cro-loaded nanocarrier can be attributed to the interaction between Cro and the nanocarrier.

**Figure 9:**
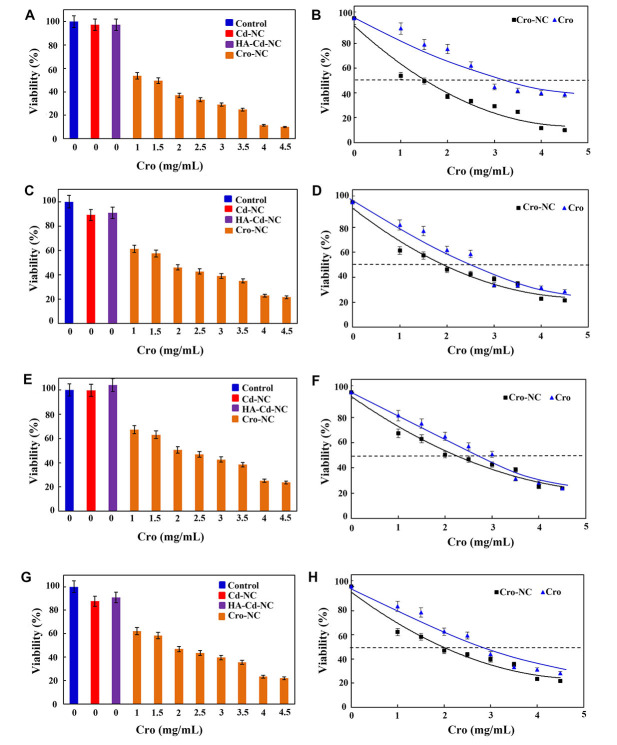
The viability assay of a panel of breast cancer cell lines in the presence of Cd-NC. (A) MDA-MB-468 treated with Cd-NC, HA-Cd-NC, and Cro-NC that was determined by MTT assay; (B) the effect of different doses of free Cro and the equivalent concentration loaded on Cro-NC on the viability of the treated MDA-MB-468 cells; (C) MDA-MB-231 treated with Cd-NC, HA-Cd-NC, and Cro-NC that was determined by MTT assay; (D) the effect of different doses of free Cro and the equivalent concentration loaded on Cro-NC on the viability of the treated MDA-MB-231 cells; (E) BT-474 treated with Cd-NC, HA-Cd-NC, and Cro-NC that was determined by MTT assay; (F) the effect of different doses of free Cro and the equivalent concentration loaded on Cro-NC on the viability of the treated BT-474 cells; (G) MCF-7 treated with Cd-NC, HA-Cd-NC, and Cro-NC that was determined by MTT assay; (H) the effect of different doses of free Cro and the equivalent concentration loaded on Cro-NC on the viability of the treated MCF-7 cells

In the next step, this nanocarrier was used for its therapeutic effect against a panel of breast cancer cell lines. In Figure 9A, it shows the percentages of the viability of the MDA-MB-468 breast cancer cell line in the usual medium (control) and in the cells treated with Cd-NC, HA-Cd-NC, and Cro-NC, as determined by MTT assay. A comparison between the viability of the MDA-MB-468 breast cancer cell line treated with different free Cro or Cro-NC concentrations are shown in Figure 9B. This Figure indicates the IC_50_ of Cro-NC was lower than free Cro. The similar data for MBA-MB-231, BT-474, and MCF-7 breast cancer cell lines are shown in Figure 9C–H. The IC_50_ of Cro-NC and free Cro in these cell lines are shown in Table 2. These data indicate the highest IC_50_ for free Cro, which was decreased after incorporation into different nanocarriers. The lowest one was observed in MDA-MB-468 cells with the highest degree of CD44.

**Table 2:** The IC_50_ of Cro in the form of Cro-NC and free Cro in a panel of breast cancer cell lines

**Cell line**	**The IC_50_ of Cro-NC (mg/mL)**	**The IC_50_ of free Cro (mg/mL)**
MDA-MB-468	1.5	3.5
MDA-MB-231	2	2.5
BT-474	2	3.5
MCF-7	2	3.0

## Discussion

The Cd-NC was synthesized with some modifications and a slight change in the pH based on the previously reported method [9]. A metal ion-protein adduct is formed by the reduction of the metal ions (Cd^2+^) at a high pH where the BSA acts as both a reducing and stabilizing agent. The characterization data indicate that HA conjugation, applied for targeting Cd-NC toward breast cancer cells, had no significant effect on the Cd-NC structure. This nanocluster’s safety and toxicity potential was studied both *in vitro* and *in vivo*. KMNO_4_ and CdCl_2_ were used as toxic agents and positive controls. The genotoxicity study of the Cd-NC using the comet assay in HeLa cells indicated no destructive effect against genomic (high molecular weight) DNA. In addition, the oxidative stress markers were determined in the cells and showed no oxidative stress induction in the HeLa cells after treatment with Cd-NC. The *in vivo* pharmacokinetic study showed that Cd-NC accumulated in the liver of mice after 30 days and excreted from the feces, with no significant toxicity against the tissues. It was then targeted to breast cancer cells by adding HA and loaded with Cro. Application of this cargo for a panel of breast cancer cell lines indicated its effectiveness in inducing cell death in these cells at a concentration lower than free Cro. It was also more effective against MDA-MB-468 than other breast cancer cell lines.

It has been shown that the fluorescence quantum yield of Cd-NC (2.86%) is good enough to be applied for live-cell imaging [9]. Here, various techniques were used to study the Cd-NC safety and toxicity against HeLa cells (as a human immortal cell line derived from cervical cancer and different from breast cancer), and in animal tissues. Then it was used as a safe carrier for Cro (an anticancer natural product) to a panel of breast cancer cell lines.

The comet assay was applied as a sensitive and rapid method for quantifying and analyzing the DNA damage in eukaryotic cells. It was previously used to investigate the genotoxicity of some NP [35]. CdCl_2_ and KMnO_4_ were positive controls to evaluate the cytotoxicity and genotoxicity of free Cd ions and induction of oxidative stress in the mentioned cells [36]. The comet assay results showed comet tail formation in HeLa cells after treatment with either CdCl_2_ or KMnO_4_, indicating the DNA strand break. However, there were no significant changes in the HeLa cells incubated with Cd-NC. After that, different parameters related to the comet size, shape changes, and DNA mobility in electrophoresis were considered. These parameters include the areas under the comet head and tail, the lengths of the comet head and tail, and the DNA% in the head and tail at different time intervals of HeLa cells exposure to a toxic concentration of KMnO_4_, different concentrations of Cd^2+^ and equivalent concentrations of Cd-NC. Furthermore, the head-to-tail ratio was determined for all parameters, including area, length, and DNA%. Analysis of the area and length of the comets altogether or separately showed a significant and time-dependent rise due to the treatment of HeLa cells with KMnO_4_ or different concentrations of CdCl_2_. However, Cd-NC, at any concentration, had no significant effects on these cells. Analysis of the DNA% in these segments also confirmed these results and indicated no toxicity of Cd-NC in this cell line. The results also indicate that higher Cd^2+^ concentration at 48 h induced more cell DNA strand breaks. However, there were no significant changes in the presence of Cd-NC, indicating no genotoxicity of this nano preparation of Cd against this human cell line.

In addition, the oxidative stress induction was studied in the HeLa cells after treatment with the mentioned ligands. The amounts of GSH and MDA as two important oxidative stress indicators [33], as well as the specific activity of the enzymes involved in the antioxidant defense system (CAT, GPX, and SOD), were determined in this cell line after 12 h, 24 h, and 48 h treatment with different concentrations of Cd^2+^ and equivalent concentrations of Cd-NC or toxic dose of KMnO_4_. The results indicated that in contrast to Cd^2+^ and KMnO_4_, there was no oxidative stress induction in the HeLa cells after treatment with Cd-NC.

Cd^2+^ intoxication due to occupational as well as environmental exposure in humans and due to experimental exposure in animals has been extensively investigated [37–39]. Since Cd is involved in the structure of the newly designed nanocluster, its possible toxicity was investigated in BALB/c mice as an *in vivo* model. At first, its biodistribution was studied by measuring Cd^2+^ using a FAAS method in mouse tissues. Since Cd^2+^ toxicity occurs in animals in both acute [40] and chronic conditions [41], these measurements were performed in short- (1 day), medium- (7 days), and long- (30 days) terms. In addition, the effect of Cd-NC on body weight, oxidative stress markers, and the antioxidant defense system was studied in BALB/c mice. As the data show, among the studied tissues, liver concentration of Cd was more than the others at all-time intervals of Cd-NC administration to mice. It was maximally about 2.7 μg/mg of liver tissue. These data indicate the distribution of Cd after 24 h was in the following order: liver > kidney > spleen > lung > heart. After that, it decreased gradually in most tissues, except the liver, which was increased up to the last day of the experiment. It has previously been shown that the liver is the primary site for Cd intoxication [42–45], and the accumulation of more than half of Cd in the liver leads to decreased Cd bioavailability to other sensitive organs such as the kidney [46, 47]. Here, a redistribution of Cd-NC was observed from other tissues to the liver through the bloodstream and an increase in its hepatic concentration after 30 days. Thus, although the administered Cd-NC was relatively high, 5 mg/kg body weight, Cd accumulation in the tissues other than the liver, was meager. It has been shown that toxic metals are safe in atomic and non-ionic forms involved in the structure of nanoclusters [9, 10, 48]. In addition, because of the rigidity of this structure, the BSA used in the Cd-NC structure was not destroyed and metabolized in the body. Hence, it was recirculated intact into the liver and was gradually excreted in the feces.

In parallel, the tissue samples were microscopically examined by a pathologist. As the pictures show, no significant pathologic changes were observed in the mice’s kidney, spleen, lung, and heart tissues after thirty-day treatment with Cd-NC. A slight change in the liver indicates the cytoplasmic vacuolation of all groups, including the control, possibly due to fixative inconsistencies [49].

Previous studies on the toxicity evaluation of Cd ion have indicated that hepatic toxicity of Cd ion mediated throughout the depletion of GSH and lipid peroxidation (increased MDA) due to the formation of reactive oxygen species (ROS), as well as inhibition of the antioxidant enzymes [50–52]. In addition, the pretreated mice with different NP after exposure to CdCl_2_ (10 mg/kg) for three weeks have shown a significant increase in lipid peroxidation and a significant decrease in GSH, GST, CAT, and SOD levels in all tissues [53]. Since it was shown that Cd mainly accumulated in the liver after Cd-NC administration, the possibility of liver damage was examined with more scrutiny. For this purpose, the liver tissue’s oxidant/antioxidant profile was examined. The *in vivo* data indicate no significant differences in the GSH and MDA levels between the control and Cd-NC treated mice in any groups or at any time course after exposure. In addition, no statistically significant differences were observed in the liver CAT and GST specific activities between control and Cd-NC treated mice. The only change was the specific activity of SOD in the liver at 24 h and after 7 days of Cd-NC administration, which was returned after 30 days of treatment. Although all of the data mentioned above indicate the safety of this nanocluster for mice, it must be confirmed in humans in the near future.

In the next step, the loading (in terms of entrapment efficiency) and releasing of Cro, as a natural carotenoid with the known anticancer property, was investigated on this nanocluster. The results indicated the acceptable yield of Cro loading and releasing efficiency, especially at acidic pH in the cancer cells’ environment. Similarly, doxorubicin was released much easier from this nanocarrier at acidic pH than neutral ones [9]. Thus, in the next step, the anticancer effect of Cro carried by this nanocarrier was studied and compared with the free Cro in a panel of breast cancer cells with different amounts of CD44, a steroid hormone receptor, on the cell surface.

The CD44 is a cell surface receptor responsible for cell proliferation, differentiation, migration, angiogenesis, presentation of cytokines and chemokines, and other functions in cells. However, its high expression on the surface of some cancer cells has been attributed to the metastatic ability of these cancers [54, 55]. Thus, CD44 has been known as a marker of metastatic breast cancer, and its expression is higher in the MDA-MB-468 [55]. HA has been originally introduced as a ligand of CD44 [54]. It has previously shown the entrance of HA-Cd-NC into the MCF-7 breast cancer cell, while it could not enter the HEK-293 kidney cells [9]. The reason is possible because of the presence of CD44 on the surface of MCF-7, which mediates receptor-dependent endocytosis through interaction with HA. However, HEK-293 lacks the CD44. Here, it was examined and compared the potential of the HA-Cd-NC to deliver Cro into different breast cancer cell lines with different amounts of CD44 on the surface and cell death induction in these cells.

As the results show, the percentages of viable cells decreased by increasing the concentration of Cro loaded on this nanocarrier. In addition, Cro-NC was more effective than free Cro and induced cell death at lower Cro concentrations. For example, in the MDA-MB-468 breast cancer cell line, the IC_50_ of free Cro was about 3.5 mg/mL, which is compatible with the previous report on this cell line [56]. However, the IC_50_ of Cro carried by this NC at 24 h was 1.5 mg/mL. This significant difference refers to the targeted transfer of Cro to the vicinity of the cell membrane by the nanocarrier and release on the site of action. These breast cancer cell lines contain CD44 as a surface receptor [57]. However, the expression of CD44 on the surface of MDA-MB-468 was higher, and this cell line has a more metastatic nature than other cell lines used here. Thus, the differences in the IC_50_ of Cro in the free form and those carried by this nanocarrier were more in MDA-MB-468, a metastatic breast cancer cell with more Cd44 on the surface, than the other breast cancer cell lines. These results remain to be confirmed in the *in vivo* studies in the next project.

In conclusion, the safety and nontoxicity of Cd-NC was observed in both HeLa cells and mice. Compared with Cd^2+^ applied in the form of CdCl_2_, no genotoxicity and oxidative stress induction occurred due to the equivalent or higher concentrations of Cd-NC in both *in vitro* and *in vivo* studies. These results indicate that Cro became more toxic against all four breast cancer cell lines when Cd-NC-HA was delivered. Applying this nanocarrier, which targets breast cancer cells, requires a lower concentration of anticancer compounds such as Cro for apoptosis induction. In other words, this nanocluster carried and introduced Cro into the target cell by a receptor-dependent endocytosis mechanism. It is finally released into the cancer cells and induces cell death in these cancer cells.

## Abbreviations

BSA: bovine serum albumin
CAT: catalase
CD: circular dichroism
Cd^2+^: divalent cationic cadmium
Cd-NC: cadmium nanocluster
Cro: crocin
DW: distilled water
EE: encapsulation efficiency
FAAS: flame atomic absorption spectroscopy
FI: fluorescence intensity
FT-IR: Fourier transform infrared
GPX: glutathione peroxidase
GSH: glutathione
GST: glutathione s-transferase
HA: hyaluronic acid
i.p.: intra-peritoneal
IC_50_: half-maximal drug inhibitory concentration
KMnO_4_: potassium permanganate
MDA: malondialdehyde
NP: nanoparticles
PBS: phosphate buffer saline
SEM: scanning electron microscopy
SOD: superoxide dismutase

## Declarations

### Availability of data and materials

The raw data supporting the conclusions of this manuscript will be made available by the authors (S. Zahra Bathaie, bathai_z@modares.ac.ir), without undue reservation, to any qualified researcher.

### Copyright

© The Author(s) 2024.
